# Effects of Physical Activity, Exercise and Sport on Executive Function in Young People with Attention Deficit Hyperactivity Disorder: A Systematic Review

**DOI:** 10.3390/ejihpe12010006

**Published:** 2022-01-14

**Authors:** Felipe Montalva-Valenzuela, Oscar Andrades-Ramírez, Antonio Castillo-Paredes

**Affiliations:** 1 Departamento de Educación, Escuela de Pedagogía en Educación Física, Facultad de Educación, Universidad de Las Américas, Santiago 8370035, Chile; felipe.montalva@edu.udla.cl; 2 Grupo CEAyS, Escuela de Pedagogía en Educación Física, Facultad de educación, Universidad de Las Américas, Concepción 4090940, Chile; oandrades@udla.cl; 3 Grupo AFySE, Investigación en Actividad Física y Salud Escolar, Escuela de Pedagogía en Educación Física, Facultad de Educación, Universidad de Las Américas, Santiago 8370035, Chile

**Keywords:** ADHD, children, adolescents, complementary treatment, sport therapy

## Abstract

Attention deficit hyperactivity disorder (ADHD) is a neurodevelopmental disorder that negatively affects the inattention, disorganization, and/or hyperactivity–impulsivity in children and adolescents who suffer from it, included cases being reported that continue into adulthood. This disorder impairs social, academic, emotional, psychological, and health system functioning due to its high cost of treatment. The present systematic review aims to analyze the effects of physical activity, exercise, and sports on the executive function in children and adolescents diagnosed with ADHD through the scientific literature. The results show that the practice of physical activity, exercise, or sport produces improvements in executive function in children and adolescents diagnosed with ADHD, mainly through aerobic exercise. A 20 min session of physical activity, sport, or exercise leads to improvements in executive functions in children and adolescents with ADHD. It can be concluded that the practice of physical activity, exercise, and sport generate improvements on executive functions in children and adolescents with ADHD, mainly through aerobic exercise.

## 1. Introduction

Attention deficit hyperactivity disorder (ADHD) is a neurodevelopmental–mental disorder; its symptoms are disorganization and/or hyperactivity–impulsivity [1]. The worldwide prevalence of this neurodevelopmental disorder is 6% [2], with a predominance of diagnoses in boys compared to girls, specifically, a prevalence of one girl for every three boys [3]. This disorder has a negative impact on social, academic, emotional, and psychological functioning and generates high costs to society and the health system [4], where 60% of the cases of subjects diagnosed with ADHD continue into adulthood [5], given that sensory difficulties may be part of the ADHD phenotype [6].

Patients with ADHD have been shown to perform less well on some tests measuring core executive functions [7,8]. For example, compared to control groups, ADHD patients have lower performance on tasks involving inhibition [9]. This results in high difficulty in regulating and controlling attention processes, impulses and hyperactivity, emotions, following instructions, and socializing in familiar or educational settings, and problems in task completion [10,11]. On the other hand, working memory is impaired in ADHD, leading to problems in retrospective perception, awareness and mastery of time, foresight, and the ability to imitate novel behavior [12]. In addition, deficits in cognitive flexibility [13], planning, and organization [14,15] have been reported.

It has been shown in a particular way that moderate to intense aerobic exercise is a beneficial intervention for the management of symptoms [16], being able to benefit executive functions and control of attention in children with ADHD [17]. Additionally, acute, and chronic exercise could be beneficial to behavior, socio-emotional level, and cognitive functions [18]. In addition, mindfulness interventions have been used, which would allow a reduction in ADHD symptoms [19], the creation of common bonds between parents and children [20], and a reduction in certain behavioral symptoms [21], allowing these effects to be transferable at the family level and social [22]. The use of other complementary interventions is suggested to produce improvements in the symptoms of ADHD, such as the practice of the use of physical sports activities that allow a reduction of inattention and hyperactivity in children and adolescents diagnosed with ADHD [23], allowing an improvement in quality of life [24].

Physical activity allows the prevention of the progression of chronic health problems and some disabilities [25]. Furthermore, research suggests physical activity as a solution for its benefits on cognitive performance and behavior management in children with ADHD [26,27]. In addition, improvements in neurobehavioral functions are reaffirmed, allowing a reduction in impulsivity and hyperactivity, an improvement in attention, and the general performance of executive functions [28,29,30,31]. A relationship has been found between increased levels of physical activity and alleviation of ADHD characteristics [32]. Furthermore, the neurophysiological effects induced by physical activity are opposed to the pathological effects produced by ADHD [33]. Therefore, this systematic review aimed to analyze the effects of the practice of physical activity, exercise, or sport on executive functions in children and adolescents diagnosed with ADHD through the scientific literature.

## 2. Materials and Methods

### 2.1. Search Strategy

The following review is based on the PRISMA guide [34]. A search was conducted in the databases: PubMed, Scopus, WoS, and SciELO, with the following search strategy: “Children”, “Adolescent”, “sport”, “Physical activity”, “exercise”, “ADHD”, “Attention-Deficit/Hyperactivity Disorder”, “Attention-Deficit with Hyperactivity Disorder”, “Executive Function”, articles in English and Spanish (Table 1).

### 2.2. Selection Criteria

**Figure 1:**
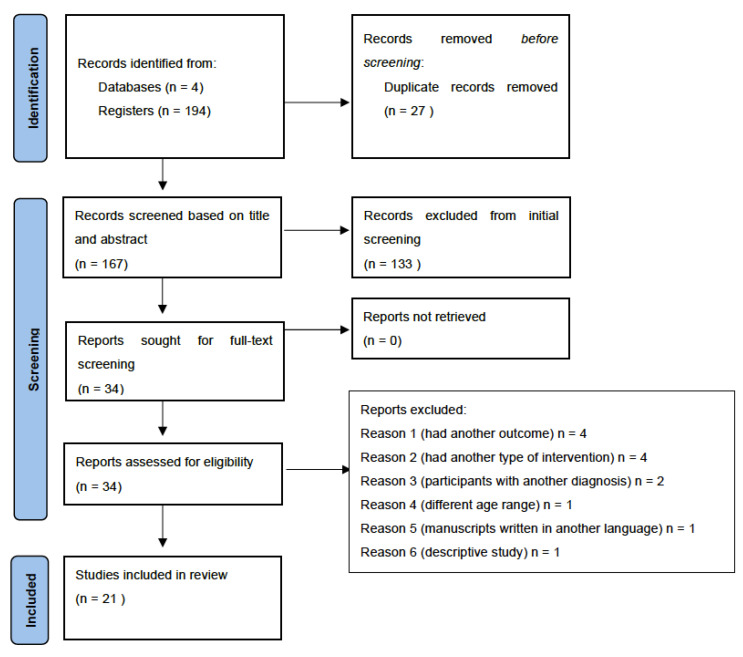
PRISMA flow chart. A total of 194 studies were found in the databases, from which 21 studies were included in this review (Figure 1).

For the analysis, studies up to August 2021 were included. Studies were included on reported interventions carried out on children and adolescents from 5 to 18 years of age diagnosed with ADHD. Exclusion criteria were subjects younger than 5 years and older than 18 years, subjects who were athletes, or subjects diagnosed with another disorder or syndrome. Research performed that had (1) another outcome, (2), another type of intervention, (3) participants with another diagnosis, (4) different age range, (5) manuscripts written in another language, (6) descriptive study, (7) letters to the editor, book chapters and conference proceedings (8) nutritional interventions, (9) literature reviews, narratives, and systematic reviews with and without meta-analysis were also excluded (Figure 1).

### 2.3. Assessing the Quality of the Selected Articles 

The risk of bias of the selected articles was scored using the Physiotherapy Evidence Database scale (PEDro) [35,36]. This scale presents 11 criteria on the internal validity and presentation of the statistical analysis, awarding one quality point to the study if it meets the criteria described and zero points if it does not [37]. The first criterion does not add up to a score (Table 1). For the selection of articles, three criteria were used (selection, comparability, and results): Interventions that received a score of eight to ten are of high methodological quality, those who received a score of four to seven are considered to be of moderate quality, and those who received a score of three to one are of low quality.

## 3. Results

### 3.1. Overall Results

PEDro scale criteria: 1: The selection criteria were known. 2: The allocation of the participants to the groups was random. 3: The assignment was hidden. 4: The groups were similar for the most relevant predictive indicators. 5: All participants were hidden. 6: All therapists for the intervention were concealed. 7: The advisers who measured at least one relevant outcome were concealed. 8: Relevant results were obtained over 85% of at least one of the most important results. 9: All outcomes of participants who completed the intervention were reported, for at least one key outcome. 10: There was a statistically significant comparison between groups for at least one key outcome. 11: The intervention revealed point and variability measures of at least one key outcome.

Of the 21 investigations that met our selection criteria, 6 investigations carried out interventions through physical activity, 12 investigations through exercise, and 3 of them based on sports (Table 2). Regarding the instrument used to confirm the diagnoses, seven of the investigations used the DSM-IV, three of the investigations used the ICD-10, four of them used the DSM-V, one of them used the DSM-IV/Chinese version of the ADHD test, one used the Chinese version of the ADHD test, and five investigations used the test to confirm the diagnosis is not available.

Articles with a score of 8 to 10 were considered of high methodological quality, those with a score of 4 to 7 of moderate quality and those with a score of less than 4 of low quality. The score obtained by the articles according to the PEDro scale indicates that the 21 articles obtained the following scores: eleven of the 21 articles obtained 5 points; seven of the 21 articles obtained 6 points; two of the 21 articles obtained 7 points and only one of the 21 articles obtained 8 points.

### 3.2. Physical Activity and Executive Functions

Regarding the physical activity intervention through exergaming [38], 51 children participated (8–12 years), 30 min, 3 days a week, for 8 weeks, through “Shape UP” (composed of six workouts, using the “Beatmaster Training Quest” with the following exercises: “Waterfall Jump”, “Stunt Run”, “Derby Skate”, “Knee up splash”, “Volcano Skate”, and “Slalom Grove”) for Xbox Kinect, showed improvements in executive functions, specifically in reaction times and inhibition. Improvement in the inhibition task was measured via a modified “Simon Task” (*p* = 0.049; d = 0.58) and reaction time via a modified “Flanker” task, (*p* = 0.029; d = 0.65), where intervention participants showed faster test-solving performance. In conclusion, exergaming could serve as an individualized intervention at home in the future. However, to maximize the benefits and make exercise games a valuable adjunct to the treatment of children with ADHD, personalized exercise games are needed.

Ziereis and Jansen [39] carried out an intervention where a total of 43 children diagnosed with ADHD (32 boys and 11 girls) between the ages of 7 and 12 participated (M = 9.45, SD = 1.43) in a 60 min session for 12 weeks composed of the following exercises per week: (1) catching, throwing, and bouncing; (2) balance training; (3) acrobatics; (4) aiming and throwing; (5) tennis; (6) slacklining; (7) juggling; (8) beach volleyball and handball; (9) juggling; (10) slacklining; (11) coordination exercises; and (12) throwing and catching. They used to measure executive functions the digit span (forward/backwards) and letter-number sequencing task of the HAWIK—IV, where there were three significant main effects of time for the following variables: in the short-term, effects of executive functioning a significant main effect of time for variable capture and aim is shown (*p* < 0.05). No other significant results were obtained. Long-term effects of executive functioning show several significant time results for the following variables: (1) WM index-score (*p* < 0.001), (2) range digits forward, (*p* < 0.001), and (3) sequence of letters–numbers (*p* < 0.05). In addition, we found significant group–time interactions for the variables: (1) WM score index (*p* < 0.001), (2) digits towards back (*p* < 0.05), and (3) letter–number sequencing *p* < 0.01. The analysis of the safety of the groups yielded two significant interactions in the group time for the variable and target capture (*p* < 0.01) and for the variable total score M-ABC (*p* < 0.001). Individual group comparisons were analyzed separately using post hoc Tukey’s tests. These tests did not show significant differences between EG1 and EG2, EG1 and CG, and EG2 and CG for all related motor variables. These findings support the hypothesis that long-term PA has a positive effect on executive functions of children with ADHD, regardless of the specificity of the PA. The outcomes indicated that regular PA can be used as a complementary or alternative non-pharmacologic treatment for ADHD.

Piepmeier et al. [40] involved 32 adolescents (12 women and 20 men, 10.75 ± 2.27 years), divided into a group with ADHD that consisted of 14 subjects (five women and nine men, 10.14 ± 1.96 years) and a non-ADHD group consisting of 18 subjects (7 woman and 11 men, 11.22 ± 2.43 years). This 30 min intervention occurred 2 days per week (60–80 RPM) on a recumbent cycle ergometer. Executive functions were measured using the Stroop test, where a significant result was obtained for the condition (*p* = 0.04), so that the participants used less time to complete the test in the exercise condition (33.71 ± 1.33 s) than in the no-exercise condition (35.58 ± 1.24 s). A main effect for the test component yielded a significant result (*p* < 0.01), and follow-up analyses showed that participants spent less time completing Part A (Word) (26.95 ± 0.70 s) and Part B (Color) (28.90 ± 1.28 s), because Part C (Word/Color) (48.08 ± 2.07 s) used more time. This research concluded that a moderate-intensity exercise session benefits processing speed and inhibitory control in adolescents diagnosed with ADHD.

The research conducted by Miklós et al. [41] involved 150 children (6 to 12 years old), classified into a non-medicated group (25 children in the exercise group and 25 children in the control group), a medicated group (25 children in the exercise group and 25 children in the control group) and a control group (25 children in the exercise group and 25 children in the control group), completing a physical activity session of 20 min at 60% and 80% of the maximal heart rate. Differences were found between the baseline and the result of the interventions in alertness, distraction, divided attention, and content flexibility (*p* > 0.05). In the physical activity group, there were significant differences in reaction time (*p* = 0.02) compared to the control group. The researchers concluded that 20 min of moderate-intensity physical activity allows significant improvements in executive function.

In the second intervention, carried out by Gawrilow et al. [42], 47 children participated, who were randomly assigned into two groups, the physical activity condition or performing a sedentary task. In the physical activity condition, they were asked to jump on a trampoline continuously for 5 min to standardize the intensity level of the exercise (if the participants stopped before 5 min, they had to jump again) compared to the participants in the sedentary condition, who, for 5 min, observed colored pictures that represented activities. Then, for both groups, a Go/NoGo task was applied for 21 min. The physical activity condition showed significant results in the inhibitory response (M = 76.83, SD = 21.68) compared to those who participated in the performing sedentary task (M = 65.54, SD = 30.10), (*p* < 0.05). The running tests showed the same pattern (errors of commission and omission in the complete task) the participants in the physical activity condition obtained significantly fewer errors (M = 32.78, SD = 26.61) than those in the sedentary condition (M = 46.08, SD = 35.28), (*p* < 0.05). There were no significant differences in perceived commitment and task difficulty between participants in both conditions. The research concluded that the application of programs to increase the level of physical activity could improve executive function.

On the other hand, the study of Benzing et al. [43] included 46 subjects between the ages of 8 and 12, of which only 46 completed the study, who were assigned to the exergaming group (*n* = 24) and the control group (*n* = 22). The exergaming group exercised through the video game “Shape Up” (Ubisoft, Montreal, Canada), using the XBOX Kinect (Microsoft, Redmond, WA); the participants interacted with the game through their body movements. For 15 min, participants in the physical activity condition played “Shape UP”, completing “Beatmaster Training Quest” with its six different exercises: (1) “Waterfall Jump”; (2) “Stunt Run”; (3) “Derby Skate”; (4) “Squat Me To The Moon”; (5) “Volcano Skate”, and (6) “Slalom Grove”. The control group watched a documentary report on mountain races for 15 min. All participants in the exergaming group for 14 min were in the range of moderate to vigorous intensity; in addition, the exergaming group had more cognitive challenges (*p* = 0.003) and awakeness (*p* = 0.008) compared to the control group. No significant differences were detected in updating (*p* = 0.482; d = 0.20), nor in the accuracy scores of inhibition and switching (p > 0.05). Regarding ADHD symptoms and general psychopathology, significant effects were detected on the total global index score (*p* = 0.022; d = 0.68), whereas no significant effects were detected on the DSMIV-TR symptom scales (p > 0.05). Regarding motor ability performance, after the interventional period, the exergaming group showed a significantly better total performance than the control group (*p* = 0.008; d = 0.80). When looking at the single test items of the German Motor Test, a significant effect was found. The researchers concluded that an acute physical activity intervention through exergaming could improve function in children with ADHD.

In relation to the physical activity interventions, improvements were observed in inhibitory control, processing speed, attention, cognitive flexibility, and executive functions in general through programs based on “exergame”, “ball work”, “balance work”, “coordination”, and “trampoline work”, among others.

### 3.3. Exercise and Executive Functions

A cycle ergometer [44] followed by 20 min of coordinative exercise assessed executive functions using the “Flanker” task, before and after the intervention, and an improvement in inhibitory control and selective attention was reported. In general, there was a main effect of congruence on reaction time (*p* < 0.001) and precision (*p* < 0.001), showing shorter reaction time and higher precision for consistent tests compared to incongruous tests. For the reaction time, the main effects of group (*p* = 0.005) and time (*p* < 0.001) were found. η^2^ = 0.44, indicating shorter reaction times in healthy comparisons compared to children with ADHD and in the post-test compared to the pre-test. Furthermore, there was a time and condition interaction for reaction time (*p* = 0.001), replaced by a time x condition x group interaction (*p* = 0.023). No additional significant interactions were observed for the reaction time and accuracy. The researchers concluded that an exercise session produces improvements in inhibitory control and attention. However, they point out that an aerobic exercise session could be more effective than coordinative exercise for an improvement in inhibitory control.

Memarmoghaddam et al. [45] performed a study that included exercise for 24 sessions of 90 min for 8 weeks; the program was composed of 15 min of warm-up, 25 min of goal-directed exercises such as using a table tennis racket and balls (throwing the ball into a basket, keeping the ball on the racket while walking, among others), 10 min of station training (two types of training), 15 min of progressive treadmill running, 15 min of ball games (football and basketball, among others), and 10 min of returning to calm. All components measured in the Go-No-Go test were better in the experimental group compared to the control group. To investigate the effect of the exercise program on the Go-No-Go test, a multivariate analysis of covariance (MANCOVA) was used. The results of this test showed that the training program presented in this study had a significant effect on the Go-No-Go test (Wilkes (*p* = 0.001)). The partial eta squares were equal to 0.703 and explained 70% of the variance in the behavioral inhibition test. To determine the effectiveness rate of exercise on the components of the Go-No-Go test, ANCOVA was used. The results of the ANCOVA test showed that the exercise program had the greatest impact on the No Go-True number (*p* = 0.001) and the No Go-Error number (*p* = 0.001), being more effective than True RT (*p* = 0.001), Error RT (*p* = 0.002), the Go-True number (*p* = 0.003) and the Go-Error number (*p* = 0.003), respectively (*p* < 0.05). The researchers concluded that the practice of exercise allows an improvement in executive function.

Silva et al. [46] performed an exercise intervention including 56 subjects, of whom 28 had ADHD and 28 were healthy, and both groups were aged 10–16 years. To carry out the research, experimental groups were divided as follows: an experimental group EF (GE-EF) with 14 subjects and an experimental group (GE) with 14 subjects, both groups with subjects diagnosed with ADHD, and a control group EF (GC-EF) composed of 14 subjects and a control group (CG) composed of 14 subjects, both groups composed of healthy subjects. The proposal consisted of an intense exercise session (18 times on different days and at the same time) consisting of a 5 min relay race where balloons and plastic bags were used; then, after a 5 min rest, they played the computer game “Raiders of the Lost Treasure”, which assessed the level of attention for the GE-EF and GC-EF groups, and “Raiders of the Lost Treasure” tested their attention capacity in the GE and GC. A 30.52% improvement was obtained in the performance for the GE-EF and a 40.35% in the performance of the CG-EF. In addition, the Kruskal–Wallis test was used showing significant differences between groups (*p* < 0.0001). The researchers concluded that the practice of intense exercise can produce improvements in attention and help in school performance in children with ADHD.

Chou and Huang’s [47] exercise intervention was composed of 49 subjects aged 8 to 12 years (10.5 ± 1.05 years), of which 24 were part of the exercise group (19 boys and 5 girls, 10.71 ± 1.0 years) and 25 were part of the control group (19 boys and 6 girls, 10.30 ± 1.07 years). The exercise intervention consisted of yoga for 40 min per session, two days a week, for 8 weeks, reporting improvements in executive functions, specifically in the precision rate and reaction times, measured by the determination test, which yielded a main effect of time (*p* = 0.026). The results indicated a higher precision rate in the post-test, compared to the pre-test, and a significant interaction of group by time was also reported (*p* < 0.059). In this way, it was shown that the yoga exercise group obtained a more precise response in the post-test than the control group (*p* < 0.001; d = 1.09), with no group difference observed in the pre-test. Additionally, the exercise group obtained a higher response precision after the yoga intervention (*p* < 0.001; d = 1.22), while no change in the response accuracy was found for the control group (*p* < 0.263). Finally, the researchers concluded that an intervention through yoga could be used as an alternative treatment to improve attention and inhibition in children with ADHD.

Chang et al. [48] performed exercise research involving 27 participants aged 5–10 years (23 boys and 4 girls, 8.44 ± 8.29); exercise group = 14 (10 boys and 4 girls, 8.19 ± 7.65 years); control group = 13 (13 boys, 8.78 ± 8.33 years). The program of two 90 min aquatic sessions per week for 8 weeks consisted of a 5 min warm-up, 40 min of moderate-intensity aquatic aerobic exercise, 40 min of perceptual-motor exercise in the water, and a 5 min cool-down. Improvements in inhibitory control were observed, measured by the Go-No-Go Task test. In the Go task, concerning reaction time, there was a significant main effect of group (*p* = 0.004). However, there was no main effect of time or interaction between group and time (*p* > 0.05). It was concluded that the development of a long-term exercise program in subjects diagnosed with ADHD allows quantitative as well as qualitative improvements, the latter including enjoyment, which could allow children with ADHD to adhere to these programs for longer and in the long term. 

In the intervention by Lee et al. [49], 12 children from 1st to 4th grade diagnosed with ADHD were assigned to a combined exercise group (six children, 8.83 ± 0.98 years) and a control group (six children, 8.83 ± 0.98 years). This physical exercise research was carried out in three weekly sessions, lasting 60 min (10 min warm-up, 40 min of main exercise, and 10 min of cool-down) for a total of 12 weeks. This exercise program from Weeks 1 to 4 was performed at an intensity of 45 ~ 55% HRR or 11 ~ 12 RPE, from Weeks 5 to 8, it was performed at an intensity of 55 ~ 65% HRR or 13 ~ 14 RPE, and finally, from Weeks 9 to 12, the work intensity was 65 ~ 75% HRR or 15 ~ 16 RPE. Executive functions were measured via EEG (electroencephalogram), and EEG measures in the exercise group increased (18.22 ± 0.63 to 19.01 ± 1.41 Hz) after the 12 week session. In the non-exercise group, EEG measurements decreased (19.94 ± 2.50 to 18.90 ± 0.60 Hz) during the same period. The F4 EC measure in the exercise group increased from 18.69 ± 0.50 to 18.93 ± 0.66 Hz after the 12 week session, while it decreased from 20.43 ± 2.00 to 19.63 ± 2.18 Hz in the no exercise group. However, the changes were statistically insignificant. In the exercise group, the F4 EO measure increased significantly from 20.67 ± 1.73 to 22.30 ± 1.97 Hz (*p* < 0.05) after the 12 week session, while it decreased non-significantly from 22.45 ± 0.99 to 22.38 ± 2.74 Hz in the no exercise group. The study concluded that a 12 week combination exercise program (jump rope and ball exercises) has positive effects on EEG and frontal lobe executive function measures in children with ADHD.

On the other hand, Chang et al. [50] performed an acute exercise session of moderate intensity during 30 min of treadmill running; exercise intensity was set at 50–70% HRR of each participant’s individual HRR while the control group watched a running/exercise-related video. The 40 participants (10.43 ± 0.90 years) were assigned to two groups, the control group (20 subjects, 10.42 ± 0.87 years) and the exercise group (*n* = 20, 10.45 ± 0.95 years). The results showed improvements in inhibitory control as measured by the Stroop test and the WCST. In the study, significant improvements were found over time for the “WCST” (*p* < 0.05), the “Stroop Word” (*p* < 0.01), the “Stroop Color” (*p* < 0.01), and in the latter tests for the post test. Researchers conclude that acute exercise produces positive effects on executive function in children diagnosed with ADHD.

The work carried out by Pontifex et al. [51] consisted of 20 subjects diagnosed with ADHD (six girls), and 20 subjects (six girls) in the healthy party control. Research participants performed 20 min of sitting reading or aerobic exercise on a motorized treadmill at 65–75% of maximum heart rate. Through the results, the ADHD group showed a lower precision of the general response compared to the control group (*p* = 0.026). Later, after the exercise session, both groups showed a higher precision of the reading response (*p* = 0.011). The neuroelectric measures of the analysis of the P3 component revealed lower P3 amplitude in children with ADHD compared to the healthy party control group only for the incongruous tests of the flank task (*p* = 0.009; d = 0.91). Children with ADHD, like healthy match control children, exhibited greater P3 amplitude after exercise compared to after reading (*p* = 0.001; d = 0.8). However, no differences were observed between the groups after the single exercise session (*p* = 0.98; Cohen d = 0.01). The match control children showed improved performance after exercise on the reading comprehension and arithmetic tests compared to after the sitting reading condition (reading comprehension *p* < 0.001; d = 1.58; arithmetic *p* = 0.03; d = 1.25). Finally, the researchers concluded the practice of moderate-intensity aerobic exercise could be beneficial for children diagnosed with ADHD, allowing them improvements in neurocognitive function, inhibitory control, and academic performance.

In the second study by Hung et al. [52], 34 children diagnosed with ADHD participated. The protocol consisted of watching a video for 30 min and moderate-intensity aerobic exercise for 30 min on a treadmill, which included 5 min of warm-up, 20 min of main activity, and 5 min of cool-down. The main effects analysis revealed a significant effect condition for both sessions. Participants responded faster in the pure condition than in the mixed condition after exercise (*p* < 0.01) and post-rest (*p* < 0.01). However, no significant differences (*p* > 0.05) or mixed (*p* > 0.05) conditions were observed between the two types of sessions. The study allowed expanding the current knowledge between acute exercise and executive function in children with ADHD, which could obtain positive results in working memory.

On the other hand, the research developed by Durgut et al. [53] involved 30 children with ADHD, randomly assigned to the groups, which were “treadmill training (TT = 15)” or “vibration full-body training plus treadmill training” (TT + WBVT = 15). Both groups received treadmill training 3 days a week for 8 weeks. This protocol consisted of 45 min, divided into warm-up (10 min), moderate-intensity walks (25 min), and cool-down (10 min) in relation to work on a sinusoidal vibration platform, after TT with a 5 min rest between TT and WBVT. The effects of interventions on Stroop test, and completion times of all five sub tests significantly improved in both groups (*p* < 0.05) except STP-TBAG-4 in the TT group, with no difference between groups. The number of corrections in all five subtests significantly improved in both groups (*p* < 0.05) except STP-TBAG-3 and STP-TBAG-5 in the TT group, with no difference between groups. CRS scores improved significantly in both groups (*p* < 0.05) while the magnitude of improvement in CTRS-R/L score was greater in the TT + WBVT group compared to the TT group (*p* = 0.041). The researchers concluded from the interventions that the children with ADHD only obtained improvements in their behavior in the classroom.

The intervention carried out by Ludyga et al. [54] included 36 subjects (ADHD = 18; healthy peers = 18). Participants in both groups completed 20 min of moderate-intensity aerobic exercise on a cycling bout on an ergometer, and a physically inactive control group watched a documentary video sitting for 20 min. When comparing cognitive flexibility between children with ADHD and healthy controls, there were no significant differences between the groups (*p* = 0.163). Regarding the acute effects of exercise on task performance, the MANOVA revealed a significant multivariate main effect for the condition (*p* = 0.047), and the power to detect the effect was 0.68. Based on a more detailed examination, significant univariate main effects were obtained for the condition for the category (*p* = 0.043), fluency (*p* = 0.038), and originality (*p* = 0.045). These effects indicated higher scores after aerobic exercise compared to the control condition. The findings found by the researchers pointed out that exercise causes similar benefits for cognitive flexibility in healthy children and children diagnosed with ADHD.

In the work carried out by Chan et al. [55], 37 subjects diagnosed with ADHD participated (boys = 27; girls = 10). The intervention group included 15 boys and 6 girls, and the control group included 12 boys and 4 girls. The exercise program was developed in eight consecutive weeks of two weekly sessions (16 sessions) with a duration of 60 min divided into dynamic stretching in the warm-up (5 min), moderate-intensity interval training (20 min), perceptual-motor exercise (20 min), a dynamic group game (10 min), and cool-down (5 min). The study recorded the reaction behavior of the participants with the visual reaction time test. The SRT in the intervention group and the control group did not show significant differences (*p* > 0.05). The CRT of the whole group was reduced in the intervention group. The variability of SRT and The CRT in the whole group suggests that there are no significant differences between the pre-test and the post-test in the intervention group (*p* > 0.05). Girl’s CRT was found to be significantly reduced in the intervention (*p* < 0.05). Finally, the program developed in the research contributed to the performance of a task in children with ADHD, allowing them to be a tool for the improvement of executive function.

The exercise interventions allowed benefits to inhibitory control, attention, neurocognitive functions, academic performance, cognitive flexibility, and executive function. The programs used were cycle ergometer, ball training, one intense exercise session, yoga exercise, aquatic exercise, combined exercises (jump rope and ball), moderate-intensity acute exercise, aerobic exercise on a treadmill, and moderate-intensity interval training, among others.

### 3.4. Sport and Executive Functions

The sports intervention of Pan et al. [27] involved 32 children (6 to 12 years old), who were assigned to Group I = 16 children (8.93 ± 1.49) and Group II = 16 children (8.87 ± 1.56). This program was developed twice a week, and each session consisted of 24 sessions (12 weeks) of 70 min of table tennis. Each intervention consisted of a warm-up (5 min), motor skills practice (20 min), executive function training through the use of table tennis exercises (20 min), group games (20 min), and cool-down (5 min). This intervention produced improvements in executive functions, measured in Stroop color–word scoring time (*p* < 0.01), and compared to the control group, the exercise group performed better on the test (*p* < 0.01). The changes in the Stroop color–word score for Group I remained stable, with differences between Time 2 and Time 3 not being significant. However, Stroop’s word and color score at Time 3 differed significantly in Group II compared to Time 2 (*p* < 0.01; d = 1.55). The researchers conclude that a long-term racket sports intervention is an effective strategy to improve some cognitive and behavioral functions in children diagnosed with ADHD.

Kadri et al. [56] 2019 in their Taekwondo sports program (TKD), composed of 40 young people diagnosed with ADHD (36 men and four women), were randomly assigned to a group of TKD (*n* = 20, age = 14.5 ± 3, 5 years, 18 men and two women) or a control group (*n* = 20, age = 14.2 ± 3 years, 18 men and two women). The TKD group carried out the intervention program for the development of sport technical skills (blocking, punching, and kicking, among others) and poomse (technical forms with a particular order), which consisted of a 50 min program twice (30 min of TDK and 10 min of warm-up and cool down, for 18 weeks) of TKD. Compared to the control group, which developed athletics, handball, and gymnastics activities, during two physical education sessions per week at school. Regarding the results, there were significant differences between both groups, for the “Stroop color” test (*p* = 0.001), word color inference test (large ES = 2.16; *p* < 0.001), interference test (large ES = 1.63; *p* < 0.001), and error (large ES = −2.20; *p* < 0.001). The researchers concluded the practice of TKD could increase selective attention in adolescents with ADHD.

The research developed by Pan et al. [57] involved 60 subjects, distributed into ADHD training (*n* = 15), ADHD non-training (*n* = 15), and TD children without ADHD non-training (*n* = 30). The table tennis intervention was developed for 12 weeks and twice a week, with a duration of 70 min, distributed in warm-up (5 min), basic table tennis skills (20 min), training of executive functions through of table tennis (20 min), 20 min of group games (20 min), and calm down (5 min). Changes in effect size (ES) were obtained in the ADHD group in the Stroop color plus word test (ES = 2.07), indicating a large ES. For the Test of Gross Motor Development (TGMD-2), a large ES was obtained for the locomotor and object control variable (ES = 1.90) and (ES = 3.04), respectively. For the total Wisconsin Card Sorting Test (WCST), a large ES was obtained (ES = 1.19). We did not observe group differences both for locomotor (*p* = 0.59) and control of objects (*p* = 0.06) skills before the intervention. It was concluded that 12 weeks of table tennis exercises may have clinical relevance to executive functions in children with ADHD.

Through sports programs, benefits were obtained on some cognitive and behavioral functions, selective attention, and executive functions in general through interventions such as table tennis and taekwondo.

The 21 investigations met the selection criteria of the investigation, which demonstrated the benefits of the practice of physical activity, exercise, or sport. Of the 21 investigations, 6 developed interventions with physical activity programs [38,39,40,41,42,43], 12 investigations developed interventions with exercise programs [44,45,46,47,48,49,50,51,52,53,54,55], and only 3 studies developed sports programs [27,56,57].

## 4. Discussion

Therefore, the purpose of this study is to analyze the effects of the practice of physical activity, exercise, or sport on executive functions in children and adolescents diagnosed with ADHD through the scientific literature.

In relation to the physical activity interventions, programs were found using “exergame” [38,43], “work of coordination, balance, and acrobatics, among others” [39], “cycle ergometer” [40], “20 min session of physical activity” [41], and “jumping on a trampoline” [42]. All these programs had a minimum duration of 20 to 60 min per work session.

The exercise interventions developed programs through “cycle ergometer” [44], “exercises combining ball work, table tennis, and treadmill running” [45], “moderate to vigorous and intense exercise” [46,49,50,51,54], “yoga exercises” [47], “aquatic exercise” [48], “treadmill” [50,52], “treadmill training or the whole-body vibration training” [53], “dynamic stretching, moderate-intensity interval training, and perceptual-motor and dynamic games” [55], with a minimum duration of 20 min and a maximum of 90 min. Finally, the investigations that used sports programs were through “table tennis” [27,57] and “taekwondo” [56], with a duration of 50 to 70 min.

The present systematic review made it possible to demonstrate the benefits of practicing physical activity on executive function through different programs. An investigation showed beneficial results on executive function through the practice of physical activity, establishing an association between aerobic fitness and executive function through the performance of cognitively challenging physical activity through a physical education program plus two hours of tennis-specific skills training [58]. In addition, the performance of physical exercise demonstrated the obtaining of significant results on executive function; these results are consistent with those reported in a systematic review, which concludes that the practice of acute and chronic exercise can produce improvements in executive function; there is a small effect size on inhibitory control and cognitive flexibility and moderate effect sizes on working memory [59]. Regarding the sports interventions carried out through tennis, they obtained significant results on executive function; an intervention within their conclusions indicated that table tennis allows improvements in executive function because it requires a greater commitment of cognitive flexibility [60].

While the results of all interventions report improvements in executive functions, there is heterogeneity among many of the investigations concerning the time spent in each study. On the other hand, there is also a high diversity around physical activity, exercise, and sports programs, such as yoga, running, table tennis, and swimming, among others. In addition, the difference between the tests performed to assess executive functions does not help to discern or recommend which physical activity or exercise or sports program is best for ADHD symptomatology, considering the particular characteristics in school contexts. 

Possible conditioning factors of the research are the high prevalence rate of the disorder in males, which generates very homogeneous samples in terms of gender, the lack of review or monitoring of the physical fitness or sedentary lifestyle of the participants of the interventions and the daily control of the medication used. In addition, the lack of post-intervention follow-up may raise doubts as to whether these are significant or momentary changes.

### 4.1. Limitations

The limitations of this review stem mainly from the lack of analysis of the gray literature, the consideration of the review of scientific research until August 2021. Due to the different programs implemented in the research, it is difficult to establish a comparison to determine which intervention program is more effective over another specific program between studies because the results obtained in the analyses of the research consulted are significant.

### 4.2. Future Implications

It is suggested to implement physical activity, exercise, or sport interventions with a minimum duration of 20 min per work session, complementary to a sports workshop, to improve executive functions in children and adolescents diagnosed with ADHD. In this way, it also helps the motor and social aspects, variables that are also conditioned in ADHD sufferers.

## 5. Conclusions

The present systematic review showed the positive effects on executive function through the development of programs and interventions of physical activity, exercise, or sport, which could be used as programs and/or complementary interventions to the Physical Education class, which could be implemented with due caution. Although these programs and interventions are heterogeneous, they allowed improvements in inhibitory control, processing speed, selective attention, cognitive flexibility, some cognitive and behavioral functions, academic performance, and executive functions in general in children and adolescents with ADHD.

## 6. Recommendations

According to the review, the incorporation of physical activity or aerobic exercise of moderate to vigorous intensity is recommended in addition to individual sports such as martial arts, due to the reduction of anxiety levels that they produce in adolescents and group sports due to the social component they have. With the previous antecedents, it should be considered as an alternative treatment or complementary therapy in subjects diagnosed with ADHD for the improvement of executive functions.

Research is needed on the improvement of executive functions through interventions of strength, coordination, or flexibility, as well as an evaluation of the physical condition or physical condition of the participants and a more exhaustive control of the use of medication, considering the improvement of motor skills, biological, social, and psychological aspects such as motivation and self-esteem generated by this type of intervention.

## Figures and Tables

**Table 1:** Methodological analysis of the articles (PEDro scale).

Authors	Criteria											
	1	2	3	4	5	6	7	8	9	10	11	Total
Benzing et al., 2019	Y	Y	Y	Y	N	N	N	Y	Y	Y	Y	7
Ziereis & Jansen, 2015	Y	Y	N	Y	N	N	N	Y	Y	Y	Y	6
Piepmeier et al., 2015	Y	N	N	Y	N	N	N	Y	Y	Y	Y	5
Miklós et al., 2020	Y	N	N	Y	N	N	N	Y	Y	Y	Y	5
Gawrilow et al., 2016	Y	N	N	Y	N	N	N	Y	Y	Y	Y	5
Benzing et al., 2018	Y	Y	Y	Y	N	N	N	Y	Y	Y	Y	7
Ludyga et al., 2017	Y	N	N	Y	N	N	N	Y	Y	Y	Y	5
Memarmoghaddam et al., 2016	Y	Y	N	Y	N	N	N	Y	Y	Y	Y	6
Silva et al., 2015	Y	N	N	Y	N	N	N	Y	Y	Y	Y	5
Chang et al., 2014	Y	N	N	Y	N	N	N	Y	Y	Y	Y	5
Chou & Huang, 2017	Y	N	N	Y	N	N	N	Y	Y	Y	Y	5
Lee et al., 2017	Y	Y	N	Y	N	N	N	Y	Y	Y	Y	6
Chang et al., 2012	Y	Y	N	Y	N	N	N	Y	Y	Y	Y	6
Pontifex et al., 2013	Y	N	N	Y	N	N	N	Y	Y	Y	Y	5
Hung et al., 2016	Y	N	N	Y	N	N	N	Y	Y	Y	Y	5
Durgut et al., 2020	Y	Y	N	Y	N	N	N	Y	Y	Y	Y	6
Ludyga et al., 2018	Y	Y	N	Y	N	Y	Y	Y	Y	Y	Y	8
Chan & Ho, 2021	Y	N	N	Y	N	N	N	Y	Y	Y	Y	5
Pan et al., 2016	Y	Y	N	Y	N	N	N	Y	Y	Y	Y	6
Kadri et al., 2019	Y	Y	N	Y	N	N	N	Y	Y	Y	Y	6
Pan et al., 2015	Y	N	N	Y	N	N	N	Y	Y	Y	Y	5

Note: Y = Yes; N = No.

**Table 2:** Characteristics of the investigation.

	Subjects	Objective	Intervention Type
Benzing et al., 2019	51 subjects (82.4% boys and 17.6% girls aged 8 to 12 years).	Investigate the effects of cognitively and physically demanding exergaming on executive function	Physical activity
	Exercise = 28 (10.46 ± 1.30)		
	Control = 23 (10.39 ± 1.44)		
Ziereis & Jansen, 2015	43 subjects (32 boys and 11 girls aged 7 to 12 years).	Determine whether physical activity improves cognitive performance in children with ADHD	Physical activity
	Experimental Group 1 = 13 (9.2 ± 1.3)		
	Experimental Group 2 = 14 (9.6 ± 1.6)		
	Control Group = 16 (9.5 ±1.4)		
Piepmeier et al., 2015	32 adolescents (female gender 12 and male gender 20, 10.75 ± 2.27 years)	Examine the effect of acute exercise on cognitive performance by children with and without ADHD	Physical activity
	ADHD group = 14 (10.14 ± 1.96 years)		
	Non-ADHD group = 18 (11.22 ± 2.43 years)		
Miklós et al., 2020	150 children aged 6–12 years.	Examine the effects of an intense exercise on attention and executive function	Physical activity
	Non-medicated group = 50 (25 exercise and 25 control).		
	Medicated group = 50 (25 exercise and 25 control).		
	Control group = 50 (25 exercise and 25 control).		
Gawrilow et al., 2016	47 boys	Investigate the effects of a single bout of physical activity on cognitive functioning in children with ADHD	Physical activity
	(8.3 to 13.6 years)		
	(M = 10.47; SD = 1.49)		
Benzing et al., 2018	46 children ADHD Exergaming = 24 Control = 22	Investigate the effects of an acute bout of physical activity on multiple aspects of executive functions in children with ADHD	Physical activity
	8–12 years (82.6% boys)		
Ludyga et al., 2017	36 subjects (11 to 16 years)	Investigate the acute effects of aerobic exercise and coordinative exercise on inhibitory control in children with ADHD	Exercise
	ADHD = 18 (7 girls and 11 boys)		
	Control = 18 (8 girls and 10 boys)		
Memarmoghaddam et al., 2016	36 male students (7 to 11 years)	Examine the effectiveness of a Selected exercise program on the executive function of children with ADHD	Exercise
	PA group = 19 (8.31 ± 1.29)		
	Control Group = 17 (8.29 ± 1.31)		
Silva et al., 2015	56 volunteers aged 10–16 years.	Quantify the effect of physical activity on the attention of children with ADHD	Exercise
	CG = 28 subjects without ADHD.		
	SG = 28 subjects with ADHD and no medication (30 remained).		
Chou & Huang, 2017	49 participants (38 boys and 11 girls, 10.50 ± 1.05 years).	Investigated whether a yoga exercise intervention influenced the sustained attention and discrimination function in children with ADHD	Exercise
	Exercise group = 24 (19 boys and 5 girls, 10.71 ± 1.00 years).		
	Control group = 25 (19 boys and 6 girls, 10.30 ± 1.07 years)		
Chang et al., 2014	27 participants aged 5 to 10 years (23 boys and 4 girls, 8.44 ± 8.29)	Examine whether an aquatic exercise intervention influences restraint inhibition in children with ADHD	Exercise
	Exercise group = 14 (10 boys and 4 girls, 8.19 ± 7.65 years).		
	Control group = 13 (13 children, 8.78 ± 8.33 years).		
Lee et al., 2017	12 children	Investigate the effects of exercise on neuropsychological variables of executive function with electroencephalography in children with ADHD	Exercise
	CEG = 6 (8.83 ± 0.98 years)		
	NEG = 6 (8.83 ± 0.98 years)		
Chang et al., 2012	40 Children	Determine the effect of acute aerobic exercise on executive function in children with ADHD	Exercise
	(3 girls y 37 boys, 8–13 years)		
Pontifex et al., 2013	ADHD Group = 20 (6 females), age 8–10	Examine the effects of a single bout of aerobic exercise on the inhibitory control deficits in children with ADHD	Exercise
	Healthy Group = 20 children (6 females), age 9.8 ± 0.1		
Hung et al., 2016	34 children with ADHD (1 female)	Examined the effects of an acute aerobic exercise session on task switching in children with ADHD	Exercise
	aged 10.16 ± 1.74		
Durgut et al., 2020	30 children ADHD	Compare the effects of Treadmill Training and Whole-body Vibration Training on attention and quality of life in children with ADHD.	Exercise
	age between 7–11 years		
Ludyga et al., 2020	Children ADHD = 18	Investigate cognitive flexibility and task-related heart rate variability following moderately intense aerobic exercise and after watching a video in both children with ADHD and healthy controls	Exercise
	Healthy peers = 18		
	age between 11–16 years		
Chan et al., 2021	37 children ADHD	Investigate the efficacy of an adapted physical activity program on reaction performance in children with ADHD	Exercise
	aged 8–11 years		
Pan et al., 2016	32 children (6 to 12 years).	Examine the effects of a racket-sport intervention on executive functions, motor skills, and social behaviors	Sports
	Group, I = 16 children (8.93 ± 1.49)		
	Group II = 16 children (8.87 ± 1.56)		
Kadri et al., 2019	40 young people (36 males and 4 females).	Investigate the effects of a one-and-a-half-year-long Taekwondo (TKD) intervention on cognitive function in adolescents with ADHD	Sports
	TKD group = 20 (14.5 ± 3.0 years, 18 males and two females).		
	Control group = 20 (14.2 ± 3.5 years, 18 males and two females).		
Pan et al., 2019	ADHD training = 15	Examined the effects a 12-week Table Tennis exercise on motor skills and executive functions in children with ADHD	Sports
	ADHD non-training = 15		
	TD children without ADHD non-training = 30		
	All groups age 7–12 years		
